# Irisin Protects the Human Placenta from Oxidative Stress and Apoptosis via Activation of the Akt Signaling Pathway

**DOI:** 10.3390/ijms222011229

**Published:** 2021-10-18

**Authors:** Hamid-Reza Kohan-Ghadr, Brooke Armistead, Mikaela Berg, Sascha Drewlo

**Affiliations:** Department of Obstetrics, Gynecology and Reproductive Biology, College of Human Medicine, Michigan State University, Grand Rapids, MI 49503, USA; kohangha@msu.edu (H.-R.K.-G.); armiste9@msu.edu (B.A.); bergmik1@msu.edu (M.B.)

**Keywords:** Irisin, placenta, trophoblast, preeclampsia, apoptosis, oxidative stress

## Abstract

Irisin is a newly discovered exercise-mediated polypeptide hormone. Irisin levels increase during pregnancy however, women with preeclampsia (PE) have significantly lower levels of Irisin compared to women of healthy pregnancies. Even though many studies suggest a role of Irisin in pregnancy, its function in the human placenta is unclear. In the current study, we aimed to understand key roles of Irisin through its ability to protect against apoptosis is the preeclamptic placenta and in ex vivo and in vitro models of hypoxia/re-oxygenation (H/R) injury. Our studies show that Irisin prevents cell death by reducing pro-apoptotic signaling cascades, reducing cleavage of PARP to induce DNA repair pathways and reducing activity of Caspase 3. Irisin caused an increase in the levels of anti-apoptotic BCL2 to pro-apoptotic BAX and reduced ROS levels in an in vitro model of placental ischemia. Furthermore, we show that Irisin treatment acts through the Akt signaling pathway to prevent apoptosis and enhance cell survival. Our findings provide a novel understanding for the anti-apoptotic and pro-survival properties of Irisin in the human placenta under pathological conditions. This work yields new insights into placental development and disease and points towards intervention strategies for placental insufficiencies, such as PE, by protecting and maintaining placental function through inhibiting hypoxic ischemia-induced apoptosis.

## 1. Introduction

Preeclampsia (PE) is a hypertensive disorder of pregnancy described by systemic endothelial damage in the mother. In its severe form, PE establishes clinically as early as 20 weeks of gestation. It often necessitates preterm delivery and presents a significant risk to the immediate and long-term well-being of the baby, furthermore, causing vast neonatal intensive care unit costs [1,2]. The etiology of PE is thought to originate from the placenta since, at present, the only available treatment is the removal of the placenta, requiring delivery of the baby.

In healthy pregnancy, the extra-villous trophoblast (EVT) cells of the placenta invade from the anchoring villi into the uterine wall and participate in spiral artery remodeling to establish blood supply of the placenta [3,4]. This results in a low pressure/high flow blood delivery, which maintains a steady perfusion of the placental villi and its exchange function. The hemodynamic adaptations are disturbed in severe PE pregnancies due to low EVT cell invasion and reduced spiral artery remodeling causing decreased oxygen availability for the placental villi [5]. This pathological process is accompanied with increased hypoxic and oxidative stress often resulting in significant placental apoptosis [6,7].

In the mother, endothelial dysfunction is a major contribution to maternal hypertension causing damage to the kidneys and resulting in proteinuria and renal failure [5,8]. If untreated, severe forms of PE can advance to include the hepatic and coagulation systems and damage the brain. These disease phenotypes are largely attributed to abnormal placental function resulting in an excess release of anti-angiogenic proteins secreted by the placenta [9]. Abnormal placental protein secretion such as soluble fms-like tyrosine kinase 1 (sFLT1), endoglin and others can damage the maternal endothelium and is commonly observed in PE [5].

Irisin is among many peptides that are upregulated during pregnancy and function to regulate energy homeostasis across gestation [10]. Irisin was first described in 2012 as a myokine polypeptide secreted from skeletal muscle that regulates glucose and lipid metabolism in adipose tissues in response to exercise [11]. Irisin is a secreted form of the Fibronectin Type III Domain Containing 5 (FNDC5) transmembrane protein. Studies showed that FNDC5/Irisin is expressed in adipose tissue, cardiomyocytes, the brain and other parts of the body [12,13]. Recently the interest in the molecular actions of Irisin revealed that it is not only involved in energy storage and sensing, but it has a variety of molecular functions including differentiation, inflammation, oxidative stress in different systems [14,15,16,17,18]. The inhibitory effects of Irisin on cell apoptosis was shown to occur by modulation of pro-apoptotic markers such as the BCL2 associated agonist of death (BAD), BCL2 associated X apoptosis regulator (BAX), Caspase-9 and Caspase-3 and anti-apoptotic proteins such as BCL-2 and BCL-XL [16,19,20].

The Akt signaling pathway plays an important role in regulating cell proliferation, cell migration and apoptosis inhibition [21,22,23]. Akt activation has been shown to regulate trophoblastic cell migration and invasion [24]. In the human placenta, Akt regulates trophoblast invasion through the upregulation of matrix metalloproteinase 9 (MMP-9) and tissue inhibitor of metalloproteinase-1 (TIMP-1) [25]. Under oxidative stress and hypoxia conditions, Akt is inactivated in trophoblast cells and in primary cytotrophoblast cells via Hypoxia inducible factor-1α (HIF-1α) upregulation, which subsequently triggers Glial cell missing 1 (GCM1) degradation that, in turn, inhibits migration and invasion of trophoblast cells [26,27,28,29,30]. Inactivation of the Akt pathway in human PE placentas suggests its possible contribution in PE pathophysiology and progression [31]. In addition to the crucial role in trophoblast differentiation, Akt activation has also cytoprotective effect in trophoblasts [32,33].

Irisin was shown to be reduced in the circulation of women with preeclampsia [34,35], which has led us to question its importance human reproduction and disease. Previously, we identified that Irisin modulates trophoblast differentiation through activation of the AMPK pathway [36]. However, the role of Irisin in placental function and pathophysiology, especially in pregnancy complications such as PE, is still not fully known. In our study, we hypothesized that Irisin can protect from apoptosis in the human placenta through activation of the Akt signaling pathway. To test this hypothesis, we employed Irisin treatment in preeclamptic placentas and in ex vivo and in vitro placental models of ischemic-reperfusion injury and evaluated Irisin’s effect on Akt activation and downstream apoptotic pathways.

## 2. Results

### 2.1. Irisin Rescues Villous Trophoblast Cells from Apoptosis in the Preeclamptic Term Placenta

**Figure 1:**
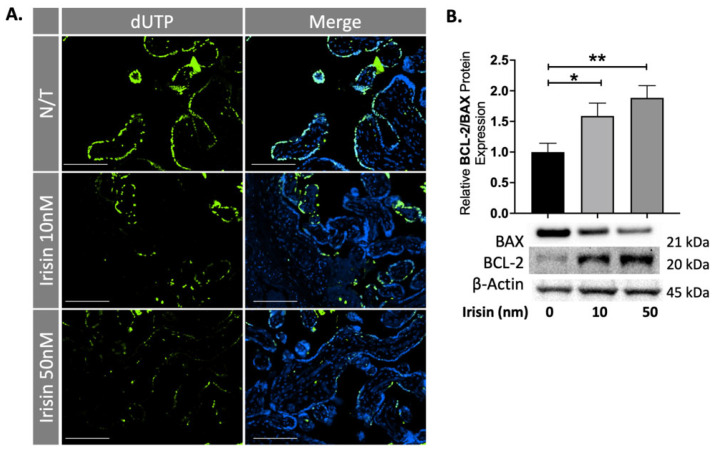
Irisin reduces apoptosis in the preeclamptic placenta. (**A**) A TUNEL assay shows a significant reduction of double-stranded DNA breaks, as measured by dUTP fluorescent molecules, when treated with Irisin. (**B**) 10 nm and 50 nm Irisin treatment in the preeclamptic placenta increased the anti-apoptotic BCL-2: pro-apoptotic BAX protein levels (n = 8). (Relative protein expression was determined by normalization to β-actin, a one-way analysis of variance and subsequent Tukey’s post hoc test to analyze differences between cohorts; * *p* < 0.05, ** *p* < 0.01. Bar plots are presented as mean ± SEM. Scale bar = 100 μm. Green staining indicates DNA strand breaks; Blue staining is for Dapi indicating the cell nucleus. dUTP, deoxyUridine TriPhosphate; PE, Preeclampsia).

The preeclamptic placenta is characterized by increased apoptosis believed to be caused by the prolonged hypoxic-ischemic stress caused by intermittent reperfusion injury during pregnancy. To test the effect of Irisin on apoptosis in the preeclamptic placenta, tissues were dissected and cultured overnight with 10 and 50 nM of Irisin and collected for protein analysis and embedded for staining. Placental sections were stained for late-stage apoptosis. DNA-strand breaks were identified by a fluorescein-based TUNEL (terminal deoxynucleotidyl transferase [TdT] dUTP nick-end labeling) assay. DNA strand breaks are thus identified by the green fluorescence as shown Figure 1A. We observed a significant reduction of apoptotic cells in the villous trophoblast cells of preeclamptic placental explants exposed to different concentrations of Irisin (Figure 1A). Downstream apoptosis markers were analyzed by western blots. There was a significant 50% and 70% increase in the ratio for the anti-apoptotic BCL2: pro-apoptotic BAX protein levels in the PE tissues treated with 10 or 50 nm Irisin, respectively, supporting the anti-apoptotic effect of Irisin in the preeclamptic placenta (n = 9, Figure 1B).

### 2.2. Irisin Significantly Decreases Apoptosis and Improves Cell Survival in 1st Trimester Human Placental Explants Stressed by Hypoxia/Re-Oxygenation

**Figure 2:**
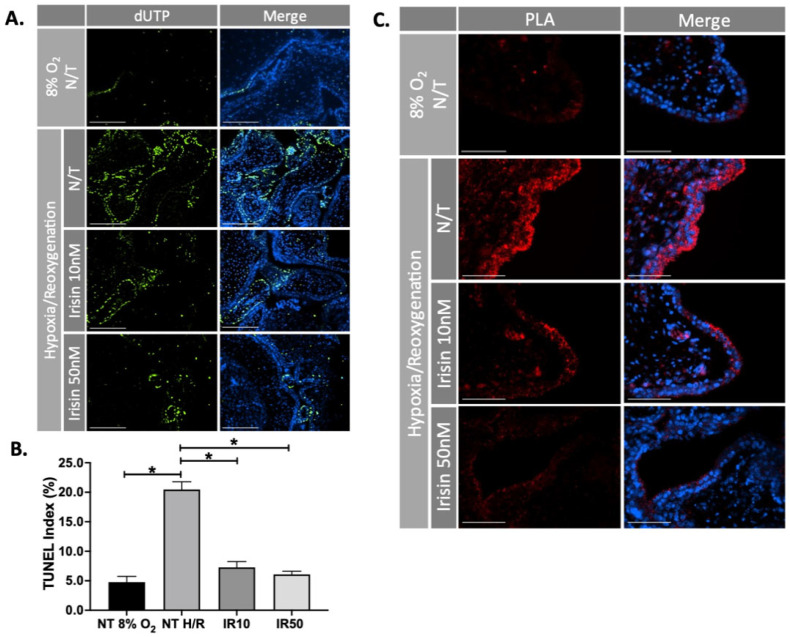
Irisin reduces apoptosis and improves cell survival in the ischemic 1st trimester human placenta. (**A**,**B**) Irisin significantly reduced the number of apoptotic cells during H/R in the first trimester placenta, as identified by a reduction of green fluorescent molecules in the TUNEL assay (n = 4). (**C**) The interaction between APAF1 and Cytochrome C (the early sign for initiation of apoptosis) indicated by red fluorescent molecules, was significantly diminished when H/R stressed first trimester placental explants were exposed to Irisin. The molecular interaction was studied by immunofluorescence in situ PLA, a novel technique that enables visualization of molecular proximity at single molecule resolution. (Significant changes in TUNEL index were measured by a one-way analysis of variance and subsequent Tukey’s post hoc test to analyze differences between cohorts; * *p* < 0.05. Scale bar = 100 μM. Green fluorescent staining indicates DNA strand breaks (**A**); Blue fluorescent staining is for Dapi indicating the cell nucleus (**A**,**C**); Red fluorescent staining indicates APAF1 and Cytochrome C interaction (**C**). Bar plots are presented as mean ± SEM; N/T, non-treatment; H/R, hypoxia/re-oxygenation; PLA, Proximity Ligation Assay; dUTP, deoxyUridine TriPhosphate).

First trimester placental explants were cultured in hypoxia/re-oxygenation (H/R) conditions (24-hours 1% O_2_ followed by 5-hours 8% O_2_), to imitate the intermittent reperfusion injury observed in the preeclamptic placenta and treated with 10 or 50 nM Irisin. Cell death was quantified by TUNEL assay. There was a significant decrease in apoptotic cells in response to Irisin treatment compared to the no treatment control (n = 4, Figure 2A,B). Early-stage apoptosis was measured by using proximity ligation assay (PLA) to investigate the interaction of Apoptotic protease activating factor-1 (APAF-1) and Cytochrome C at single molecule resolution. The binding of Cytochrome C and APAF-1 is a key step in the initiation of apoptosis to permit the formation of apoptosome complexes [37]. Irisin treatment resulted in a visual reduction of APAF1 and Cytochrome C co-localization during H/R (Figure 2C).

### 2.3. Anti-Apoptotic Effect of Irisin in 1st Trimester Placenta Explants Coincides with Akt Activation

**Figure 3:**
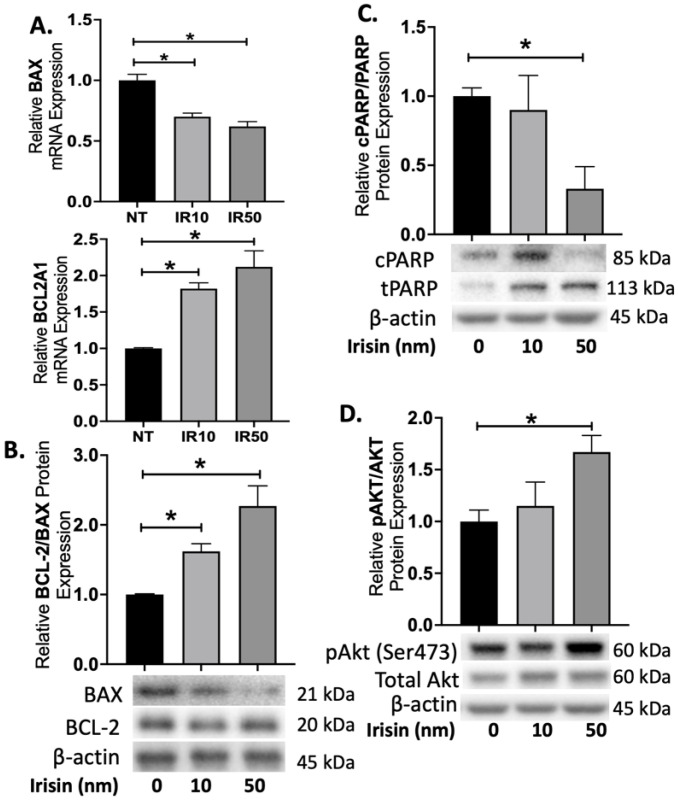
Irisin activates Akt pathway to protect against apoptosis during first trimester oxidative stress. (**A**) The gene expression of pro-apoptotic BAX decreased in presence of Irisin however the gene expression of anti-apoptotic BCL2A1 did not change with Irisin treatment (n = 3). (**B**) Protein expression for the BCL2: BAX ratio were significantly increased with Irisin treatment during H/R (n = 3). (**C**) cPARP was significantly reduced in the first trimester placenta treated with Irisin during H/R (n = 3). (**D**) Treatment of 50 nm of Irisin led to a significant increase in the ratio for phosphorylated Akt: total Akt protein levels in the first trimester placenta during H/R (n = 3). (Relative protein expression was determined by normalization to β-actin, followed by a one-way analysis of variance and subsequent Tukey’s post hoc test to analyze differences between cohort; * *p* < 0.05. Bar plots are presented as mean ± SEM. H/R, Hypoxia/Re-oxygenation, PARP, Poly (ADP) Polymerase; cPARP, cleaved PARP; tPARP, total PARP).

To evaluate how Irisin can reduce apoptosis in the ischemic first trimester placenta, we investigated expression of various proteins in the apoptotic pathways. Irisin treatment of first trimester placental explants exposed to H/R resulted in a 25–40% decrease in gene expression of the pro-apoptotic BAX, while gene expression of anti-apoptotic BCL2A1 did not significantly change (n = 3, Figure 3A). Irisin further showed a 65–105% increase in the ratio for the anti-apoptotic BCL2: pro-apoptotic BAX protein levels in the first trimester explants (n = 3, Figure 3B). We additionally investigated the levels of Poly (ADP-ribose) polymerase (PARP) cleavage in the first trimester placenta after H/R injury. PARP functions to detect DNA damage and provide base excision repair and during apoptosis [38], and its cleavage by caspases prevents the ability to undergo DNA repair. We observed a significant reduction in cleaved PARP (cPARP) compared to total PARP (tPARP) levels when treated with 50 nm Irisin (n = 3, Figure 3C). Further, we observed an increase in the ratio of phosphorylated Akt: total Akt protein levels from Irisin treatment during H/R (n = 3, Figure 3D).

### 2.4. Treatment IRISIN with Rescued Ischemic Injury in JEG-3 Cells

**Figure 4:**
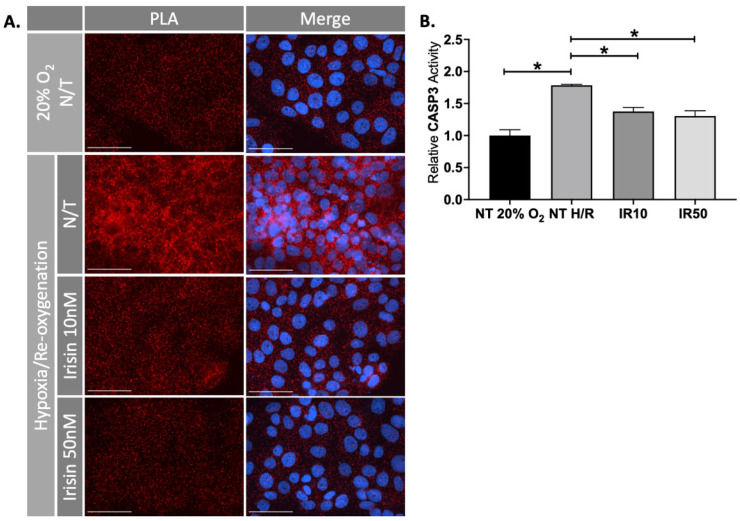
Irisin reduced apoptosis during ischemic injury in JEG-3 cells. (**A**) Proximity ligation assay (PLA) showed that the elevated APAF1/Cytochrome C interaction (apoptosis), as shown by red fluorescent molecules, under H/R condition was antagonized by Irisin in JEG-3 cells, similar to our observation in human 1st trimester explants. (**B**) This process coincided with the inhibition of Caspase 3 activity as confirmed by specific fluorogenic substrates Ac-DEVD-AMC (n = 3). (Significant changes in Caspase 3 activity were measured by a one-way analysis of variance and subsequent Tukey’s post hoc test to analyze differences between cohorts; * *p* < 0.05. Scale bar = 100 μM. Blue fluorescent staining is for Dapi indicating the cell nucleus; Red fluorescent staining indicates APAF1 and Cytochrome C interaction. Bar plots are presented as mean ± SEM. N/T, non-treatment; PLA, Proximity Ligation Assay).

In addition to our ex vivo experiments, we used the choriocarcinoma JEG-3 cell line to validate our findings in H/R conditions. PLA showed that H/R induced APAF1/Cytochrome C interaction (apoptosis) in the cell-based model, which was significantly reduced in response to Irisin treatment (n = 3, Figure 4A). Caspase 3 assists in apoptotic pathways by cleaving several cellular proteins [39]. We observed a reduction of Caspase 3 activity in the JEG-3 cells exposed to H/R after treatment with Irisin (n = 3, Figure 4B).

### 2.5. Perifosine, a Specific Akt Antagonist, Inhibited Anti-Apoptotic Effect of Irisin in JEG-3 Cells

**Figure 5:**
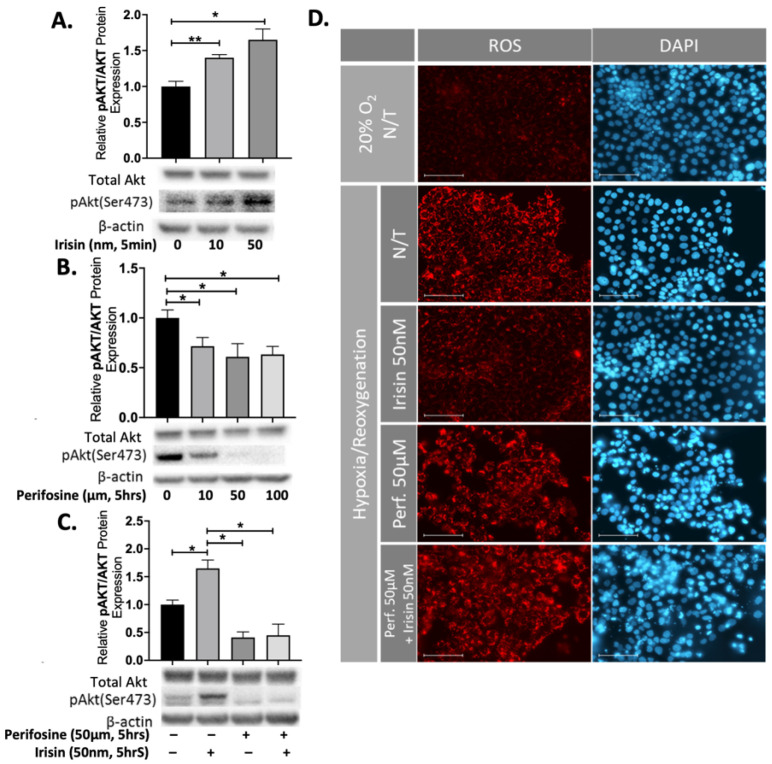
Perifosine inhibited the anti-apoptotic effect of Irisin in JEG-3 cells. (**A**) Irisin treatment increased the ratio of phosphorylated Akt: total Akt protein expression within 5-minutes of treatment (n = 3). (**B**) The ratio of phosphorylated: total Akt protein expression was significantly reduced after 5 h of increasing amounts of Perifosine (n = 3). (**C**) The promoting effect of Irisin on Akt phosphorylation was attenuated in presence of Perifosine (n = 3). (**D**) The rescue effect of Irisin on intracellular generation of ROS, shown by red fluorescence, in JEG-3 cells was blocked by Perifosine during H/R. (Relative protein expression was determined by normalization to β-actin, followed a one-way analysis of variance and subsequent Tukey’s post hoc test to analyze differences between cohorts; * *p* < 0.05, ** *p* < 0.01. Bar plots are presented as mean ± SEM. Scale bar = 100 μM. Blue fluorescent staining is for Dapi indicating the cell nucleus; Red fluorescent staining indicates ROS presence in the cells. N/T, non-treatment, H/R, Hypoxia/Re-oxygenation; ROS, Reactive Oxygen Species).

To investigate the potential role of Akt signalling in cellular response to Irisin, JEG-3 cells were incubated with 10 or 50 nm Irisin and within 5-min we observed a significant increase in the ratio for the phosphorylated Akt: total Akt protein expression levels (n = 3, Figure 5A). We incubated JEG-3 cells with different concentration of Perifosine (10–100 µM), a specific Akt inhibitor. Perifosine significantly reduced the ratio of phosphorylated Akt: total Akt protein expression (n = 3, Figure 5B). Treatment with both 50 nm Irisin and 50 µM Perifosine resulted in a significant reduction of phosphorylated Akt: total Akt protein expression levels (n = 3, Figure 5C). To further support the notion of Irisin’s apoptotic effects, we identified a significant reduction of reactive oxygen species (ROS) during H/R which was successfully blocked by Perifosine, showing that the antioxidant effect of Irisin is an Akt-dependent phenomenon (Figure 5D).

## 3. Discussion

Placental disorders such as preeclampsia often involve ischemic reperfusion injury that largely contributes to increased cell death in the placenta. In this study we provided evidence of the anti-apoptotic role for Irisin in the human preeclamptic placenta and in the human first trimester placenta and in the choriocarcinoma JEG-3 cell model during pathological conditions.

Irisin is a secreted myokine that is involved in energy metabolism. Irisin has a wide range of effects in the cell involving changes in inflammation, oxidative stress and apoptosis [16]. Exercise upregulates Irisin secretion which acts on fat tissue to induce tissue browning for the purpose of energy mobilization. Therefore, many studies have been focused on the roles of Irisin in the treatment of metabolic disorders such as obesity and type 2 diabetes, and less is known of Irisin’s roles during pregnancy.

Our study is one of the first to report anti-apoptotic features of Irisin treatment in the placenta. Evidence to support this is shown by the reduction of apoptotic cells and by an increase in the ratio of anti-apoptotic BCL2: pro-apoptotic BAX protein levels in response to Irisin-treated preeclamptic placentas and in ex vivo and in vitro model systems during ischemic reperfusion injury. The reduction of cleaved PARP induced by Irisin provides the cells an opportunity to undergo repair of DNA strand breaks in the first trimester placenta during ischemic conditions. Caspase 3 is a crucial member of apoptotic pathways by acting to cleave several cellular proteins [39] and its reduction in activity by Irisin further convinces the role for Irisin in promoting anti-apoptotic pathways. Furthermore, Caspase and BAX reduction has been described in lung injury models [40] and vascular diseases [41] in response to irisin treatment, which correlates with the findings of our study.

The increase in BAX activity during oxidative stress compromises mitochondrial membrane integrity to release Cytochrome C into the cytoplasm [42] where it is free to interact with apoptotic protease-activating factor-1 (Apaf-1) [43]. The binding of Cytochrome C with Apaf-1 initiates apoptosis signaling cascades [43]. Remarkably, we observe a dose-dependent decrease in the interaction between Cytochrome C and Apaf-1 with increasing Irisin treatment, which provides greater support to our claims that Irisin protects against apoptosis.

The Akt signaling pathway is an important intracellular signal transduction pathway with a key role in the regulation of apoptosis and cell survival [44]. Akt phosphorylation acts on several proteins including the Foxo family members, YAP, BAD and Caspase 9 to inhibit apoptosis [43]. We observed significant increases in the ratio of phosphorylated Akt: total Akt protein levels from Irisin treatment in our ischemic models. Blocking Akt activation with the Akt specific inhibitor, Perifosine, abolished the anti-apoptotic effects of Irisin, which suggests that Irisin likely acts through the Akt pathway to protect against apoptosis. Furthermore, our results correlate with additional reports in the literature. Specifically, a study by Li et al. assessed Irisin’s effect on endothelial function in apolipoprotein E-Null diabetic mice and found that Irisin treatment protected the endothelium through activation of AMPK and Akt pathways [41].

The exact signaling mechanisms on how Irisin exhibits its action are still under investigation. While we have shown that Irisin activates Akt phosphorylation at the serine 473 residue, more studies investigating members upstream and downstream of Akt are needed to further explore this pathway. A putative receptor for Irisin, αV/β5 integrin has been recently identified [45,46], which was initially characterized in the human placenta making it a prime target for Irisin signaling [47]. αV/β5 integrin has a variety of downstream targets and although it is likely Irisin will initiate these same αV/β5 integrin signaling pathways in the placenta, future studies will need to validate this process. While the placenta is known to express the full length FNDC5 protein, it is unclear if the placenta is a significant source of its secreted form, Irisin. Overall, the many physiological roles of Irisin are still under investigation and here we were able to show that Irisin has cytoprotective capabilities in the human preeclamptic placenta and models of placental disease. Future research should focus on the various sources of Irisin in pregnancy and the effects on the placenta to better understand its roles in maternal and feto-placental metabolism and disease.

## 4. Materials and Methods

### 4.1. Placental Tissue Collection

First trimester (10–12 weeks of gestation) placental tissues (n = 4) were obtained with written informed consent from healthy pregnant women undergoing elective termination of pregnancy. Term placental samples were obtained either by the Research Centre for Women’s and Infants’ Health BioBank program of Hutzel Women’s Hospital in Detroit, MI or by Women’s Health Center at Spectrum Hospital in Grand Rapids, MI, USA. The Institutional Review Board of Wayne State University and Michigan State University and approved all consent forms and protocols used in this study, which abide by the National Institutes of Health (NIH) research guidelines. Specimens were collected from pregnancies complicated by preeclampsia (n = 8; gestational age = 31–37 weeks) and were delivered either by Cesarean section or vaginal birth. Inclusion criteria for preeclampsia was in accordance with current guidelines including blood pressure >140/90 mm Hg on 2 occasions longer than 6 h apart, evidence of end-organ damage including proteinuria, with or without fetal growth restriction [48].

The collected tissues were washed and transported to the laboratory in ice-cold Hank’s balanced salt solution and were processed within a maximum of 2 h after collection. Upon arrival, tissues were snap-frozen in liquid nitrogen for further analysis. For ex vivo modeling, individual clusters of villous trees were dissected under a stereomicroscope and cultured in 1 mL of Dulbecco’s modified Eagle’s medium/Ham’s F-12 nutrient mixture (DMEM/F-12; 1:1; Life Technologies; Grand Island, NY, USA) containing 10% fetal bovine serum (FBS; Life Technologies) and 1% Gibco™ antibiotic-antimycotic. The explants were maintained overnight at either 8% O_2_ or Hypoxia/Reoxygenation (H/R) with 5% CO_2_ at 37 °C. During hypoxic exposures, the gas mixtures were balanced with N_2_. HR conditions were performed by culturing the first trimester placental explants in 1% O_2_ overnight followed by culture at 8% O_2_ and replacement of fresh medium and incubation for 5 h. For the experiments, placental explants were cultured in a medium supplemented with low (10 nM) or high (50 nM) physiological doses of active recombinant Irisin (Enzo Life Sciences, Farmingdale, NY, USA) [34,49].

### 4.2. Human Trophoblast Cell Culture

The human choriocarcinoma cell line JEG-3 was purchased from the American Type Culture Collection (ATCC). Cells were cultured in Dulbecco Modified Eagle Medium (DMEM) and Ham F12 (1:1 DMEM/F12) medium (Invitrogen, Waltham, MA, USA) containing 10% FBS and 1% Antibiotic-Antimycotic (Gibco, Amarillo, TX, USA) in a humidified incubator at 5% CO_2_. The H/R (hypoxia/reoxygenation) was performed as 1.5% O_2_ overnight followed by replacement with fresh medium equilibrated at 20% O_2_ and incubation for 5 h. To examine the effect of irisin on trophoblast differentiation, the cells were treated with 10 nM or 50 nM of recombinant Irisin (Enzo Life Sciences, Farmingdale, NY, USA). JEG-3 cells were also treated with 10 µm, 50 µm and 100 µm of Perifosine (Selleckchem, Houston, TX, USA) to inhibit Akt phosphorylation.

### 4.3. Protein Extraction and Immunoblotting

Protein extraction from tissues (20–30 mg) was performed as previously described before [50]. Protein concentration was determined with BCA™ protein assay reagent (Thermo Fisher Scientific, Rockford, IL, USA) according to the manufacturer’s instructions. Equal protein amounts (35 µg) were denatured (8 min, 95 °C) in Laemmli sample buffer (Bio-Rad Laboratories; Hercules, CA, USA) and separated using sodium dodecyl sulfate-polyacrylamide gel electrophoresis, with subsequent semi-dry transfer (Trans-Blot^®^; Bio-Rad Laboratories) to a polyvinylidene difluoride membrane. The membranes were blocked with 5% nonfat dry milk in 1× Tris-buffered saline containing 0.05% Tween-20 and were incubated overnight at 4 °C with anti-BAX (1:1000; Cell Signaling Technology, Danvers, MA, USA), anti-BCL-2α (1:1000; Cell Signaling Technology, Danvers, MA, USA), anti-cleaved PARP (Cell Signaling Technology, Danvers, MA, USA), anti-PARP (Cell Signaling Technology, Danvers, MA, USA), anti-pan-Akt (1:1000; Cell Signaling Technology, Danvers, MA, USA) and anti-phospho-Akt (Ser473) (Cell Signaling Technology, Danvers, MA, USA) primary antibodies. Subsequently, membranes were incubated with horseradish peroxidase-conjugated secondary antibodies for 1 h at room temperature and were developed with Western Lightning^®^ ECL Pro (PerkinElmer, Waltham, MA, USA). Signals were visualized using a ChemiDoc™ Imaging System (Bio-Rad Laboratories) and Image Lab Version 5.1 software (Bio-Rad Laboratories). Densities of immunoreactive bands were measured as arbitrary units by the ImageJ software (NIH, Bethesda, MD, USA). Protein levels were normalized to a housekeeping protein (β-actin; 1:4000; Cell Signaling Technology, Danvers, MA, USA).

### 4.4. Cell Death Assay

A total of 5 placentas were used for histological evaluation and quantification of apoptosis in paraffin-embedded section. A similar number of villi were dissected from all five regions mixed and then randomly picked from the pool for each experiment to avoid sampling bias. Each dissected explant contained approximately 8–10 mg tissue, four to five villi, or cultured cells. All treatments were performed in triplicates. DNA-strand breaks were detected by TUNEL (terminal deoxynucleotidyl transferase [TdT] dUTP nick-end labeling), using a fluorescein-based in situ cell death detection kit (Roche Applied Science, Indianapolis, IN, USA), according to the manufacturer’s instructions. Nuclei were counterstained with DAPI (EMD Biosciences, Billerica, MA, USA). Sections were imaged with a Nikon Eclipse 90i epifluorescence microscope (Nikon Inc., Melville, NY, USA). The apoptotic cells (TUNEL-positive nuclei) were counted at 40xX from four random fields on each section from three samples for each treatment, along with the total number of nuclei (DAPI-labeled) to calculate the percentage of TUNEL/DAPI-labeled nuclei (TUNEL index). Additional sections subjected to treatment without TdT were assessed as negative controls.

### 4.5. Proximity Ligation Assay (PLA)

Proximity ligation assay (PLA) was performed in situ using a Duolink In Situ Red Starter Kit for Mouse/Rabbit (Sigma, St. Louis, MO, USA), according to the manufacturer’s instructions. Briefly, first trimester explants were fixed in 4% paraformaldehyde, imbedded in paraffin block and sectioned onto slides for staining, followed by standard dewaxing and rehydrating conditions. JEG-3 cells were fixed and permeabilized. Cells or tissues and incubated overnight at 4 °C with primary anti-Cytochrome C and anti-APAF1 antibodies (Abcam) in pre-blocking buffer (0.05% Triton X-100 in PBS, pH 7.4). A negative control was prepared by incubating cells/tissues in blocking solution without primary antibodies. Cells/tissues were washed and incubated with rabbit plus and mouse minus PLA probes for 60 min at 37 °C. After washing, the ligation-ligase mixture was added and cells were incubated for 30 min at 37 °C, followed by an amplification step that generates a rolling circle DNA. Hoechst 33342 was used to stain nuclei. The fluorescently labeled oligonucleotides were visualized by a Nikon Eclipse 90i epifluorescence microscope (Nikon Inc.).

### 4.6. Caspase Activity Assay

The activation of caspase 3 was determined using a fluorometric substrate, Ac-DEVD-AMC (Enzo Life Sciences). JEG-3 cells were seeded at a density of 70,000 cells/well in a 6-well plate. After treatment, a nondenaturing lysis buffer was added to extract cellular protein, as described previously [51]. Total protein (35 μg) and 40 μL of substrate were added to 50 μL of reaction buffer (1% NP-40, 10% glycerol in TBS). The mixture was incubated at 37 °C for 3 h, and the fluorescence intensity was quantified using a microplate reader.

### 4.7. Fluorometric and Quantitative Evaluation of ROS Generation

To observe the basic changes of intracellular ROS, a Cellular Reactive Oxygen Species Detection Assay Kit (Abcam, Cambridge, MA, USA) was utilized as instructed by the manufacturer. JEG-3 cells were seeded in an eight-chamber slide at 10,000 cells/chamber. After a 48-h incubation, cells were washed with PBS and preloaded with 1× ROS probes for 45 min at 37 °C. Cells were then washed with PBS. Cells were examined under a Nikon Eclipse 90i epifluorescence microscope (Nikon Inc.) with appropriate filters.

### 4.8. Statistical Analysis

All statistical analysis was performed with GraphPad Prism 7.0 software. Raw mRNA and protein expressions were normalized to respective housekeeping genes or protein. All experiments were performed at least three times. A one-way analysis of variance and subsequent Tukey’s post hoc test was performed to analyze differences between cohorts. An effect was considered significant when *p* < 0.05 and is indicated with (*) on each graph. For arbitrary units, results were calculated relative to non-treatment (N/T) controls (set as 1) and presented as mean ± standard error of the mean (SEM).

## Data Availability

Not applicable.
